# Supplementation of Dihomo-γ-Linolenic Acid for Pollen-Induced Allergic Symptoms in Healthy Subjects: A Randomized, Double-Blinded, Placebo-Controlled Trial

**DOI:** 10.3390/nu15153465

**Published:** 2023-08-05

**Authors:** Kaori Yokoi, Kenichi Yanagimoto, Kohsuke Hayamizu

**Affiliations:** 1 Food Function R&D Center, Nissui Corporation, Tokyo 192-0919, Japan; yanagimoto@nissui.co.jp; 2 Laboratory of Food Chemistry, Yokohama University of Pharmacy, Yokohama 245-0066, Japan; k.hayamizu@hamayaku.ac.jp

**Keywords:** dihomo-γ-linolenic acid, allergy, unsaturated fatty acid, healthy subject

## Abstract

Dihomo-γ-linolenic acid (DGLA) is an *n*-6 polyunsaturated fatty acid that has been shown to have anti-inflammatory and anti-allergic effects in mice and cell study. To date, however, no human intervention study has examined the effects of DGLA. Therefore, we investigated the effects of DGLA on pollen-induced allergic symptoms in healthy adults. We performed a randomized, double-blind, placebo-controlled, parallel-group study comprising healthy Japanese men and women. Each subject received four 250 mg capsules providing 314 mg DGLA/day (DGLA group, n = 18) or olive oil (placebo group, n = 15) for 15 weeks. The primary outcomes, classification of the severity of allergic rhinitis symptoms (CSARS), and the Japanese Rhino-conjunctivitis Quality of Life Questionnaire (JRQLQ) served as symptom scores during the pollen season. In the DGLA group, the cedar pollen associated symptoms of sneezing and a blocked nose in the CSARS were significantly lower than those in the placebo group (*p* < 0.05, *p* < 0.01, respectively). Significant trends were observed the symptoms of runny nose in the CSARS and total symptom score (TSS) in the JRQLQ for cedar pollen (*p* < 0.1). To our knowledge, this is the first study to report the effects of DGLA in humans, and the results suggest that DGLA is effective in reducing allergic symptoms caused by pollen.

## 1. Introduction

*n*-6 polyunsaturated fatty acids (PUFAs), a series of fatty acids metabolized from linoleic acid (LA), are considered essential fatty acids that are metabolized to gamma (γ)-linolenic acid (GLA) by Δ 6 desaturase, to dihomo-γ-linolenic acid (DGLA) by carbon chain elongase, and to arachidonic acid (AA) by Δ 5 desaturase [1,2]. AA forms various inflammatory lipid mediators through the cyclooxygenase 1/2 (COX-1 and COX-2) and 15-lipoxygenase (LOX) pathways and is central to the regulation of inflammation [3]. Conversely, DGLA is metabolized by COX1/2 and LOX to a series of prostaglandins (PGs) (especially prostaglandin E1 (PGE1)) and 15-(S)-hydroxy-8,11,13-icosatrienoic acid (15-HETrE) [4], which inhibit inflammation, decrease blood pressure, enhance vasodilation, have antitumor activities, and suppress smooth muscle cell proliferation [5,6,7,8,9,10,11,12,13]. Furthermore, these DGLA-derived lipid mediators have been found to antagonize the synthesis of AA-derived lipid mediators [14]; the balance between DGLA and AA is likely an important factor influencing the body’s inflammation processes [15,16].

The physiological roles of *n*-6 PUFAs have been investigated in detail. GLA, an *n*-6 PUFA that is relatively abundant in nature and found in filamentous fungi, such as *Mortierella* and *Mucor*, and in plants, such as evening primrose and borage, has been reported to be effective in diabetes and its complications [17], rheumatoid arthritis [18], and atopic dermatitis [19]. Regarding the molecular mechanisms underlying the functions of GLA, it has been suggested that the active molecule is DGLA or a metabolite of DGLA. Furthermore, in various observational studies, abnormally low blood levels of DGLA were noted in some disease states, such as atopic dermatitis and cardiac disease [20,21,22,23]. These findings focused attention on the relationship between DGLA and disease and necessitated the study of the effects of DGLA using oils containing DGLA. DGLA is found in filamentous fungi, such as *Mortierella* species, and in various common foods, such as meat, eggs, and seafood, but in small amounts. It has not been possible to develop oils containing high concentrations of DGLA. Kawashima et al. [24] developed a fermentation method for the production of DGLA oil using a *Mortierella* strain, which enabled the preparation of large amounts of triglycerides consisting of approximately 40% DGLA fatty acids. This has enabled research on the function of DGLA, especially in atopic dermatitis in mice. In a study using mice with atopic dermatitis, the oral administration of DGLA significantly reduced the number and frequency of skin scratches and other symptoms [25,26]. Various studies, including cell studies, have shown that DGLA-mediated inhibition of the development of atopic dermatitis is mediated by reduced degranulation via prostaglandin D1 (PGD1) produced by DGLA [14,26,27,28], reduced immunoglobulin E (IgE) concentration [25], a decrease in leukotriene [29,30], and an inhibitory effect on T-cell proliferation [31].

Nasal and eye allergy symptoms are caused by the excessive production of IgE following allergen (e.g., pollen and house dust) introduction into the body [32,33]. IgE causes mast cell degranulation, resulting in histamine and leukotriene release [32,33]. Allergic symptoms caused by histamine and leukotrienes include sneezing, a runny nose, nasal obstruction, and itchy eyes, which contribute to headaches, poor concentration, and a decrease in quality of life [34]. Among nasal and eye allergies, allergic rhinitis is a global health problem with a prevalence of 10–40% worldwide, affecting 2–25% of children and up to 40% of adults [34]. Therefore, improving nasal and eye allergy symptoms is critical for improving the quality of life of many individuals. Since nasal and eye allergic symptoms (e.g., atopic dermatitis) are also caused by IgE-induced degranulation [35], a DGLA-mediated reduction in degranulation may contribute to the relief of nasal and eye allergic symptoms. Currently, three intervention studies have investigated the safety of DGLA oils for humans [36,37,38]. However, no study has evaluated the effect of DGLA on humans. Therefore, in this study, we evaluated the efficacy of DGLA oil against nasal and eye allergies in a double-blind, placebo-controlled comparative study in humans.

## 2. Materials and Methods

### 2.1. Study Design

The present study adopted a randomized, double-blind, placebo-controlled, parallel-group design.

### 2.2. Ethics

This study was pre-registered via the University Hospital Medical Information Network Clinical Trials Registry (UMIN000045540) and conducted according to the guidelines of the Declaration of Helsinki (2013) and the Ethical Guidelines for Medical Research Involving Human Subjects. The Takara Clinic Ethics Committee, Seishinkai Medical Corporation (Tokyo, Japan) approved this study (approval code: 2109-00214-0036-2C-TC).

### 2.3. Participants

The inclusion criteria were: (1) healthy Japanese adults (>20 years old) who had experienced nasal and eye discomfort due to pollen for the past two years; (2) who were not taking allergy medication (except those who occasionally taking allergy medication for nose and eye discomfort caused by pollen).

The exclusion criteria were: (1) having treatment or having a medical history of heart failure, malignancy, or cardiac infarction; (2) having a pacemaker or an implanted defibrillator; (3) at present taking treatment for any one of the chronic diseases listed as follows: arrhythmia, cerebrovascular disorder, high blood pressure, lipid disorder, hepatic disorder, renal disorder, rheumatism, diabetes, or, also, all other chronic diseases; (4) consuming functional foods or beverages at least four times a week; (5) drinking excess alcohol (average of >20 g/day as absolute alcohol intake); (6) presently taking medicines (including herbal ones) and supplements; (7) receiving long-term drug treatment (anti-allergic drugs, steroids, anti-hypertensives, antihistamines, vasoconstrictors, etc.) that may affect allergic symptoms of the nose and eyes during the trial; (8) be allergic to medications and/or intervention food related to products; (9) having a nasal irrigation habit; (10) not going out more than once a week; (11) pregnant, lactating, or planning to become pregnant; (12) be registered in other clinical trials to participate or planning to participate in other trials during this study; and (13) it was determined by the physician as inappropriate for the participant to join the study.

The study participants were recruited using a website. Of the 49 potential participants, 36 were included in this study. The 49 participants underwent the classification of the severity of allergic rhinitis symptoms (CSARS) [32,39,40] and the Japanese Rhino-conjunctivitis Quality of Life Questionnaire (JRQLQ) [32,39,40,41,42] at the time of screening. The study physicians conducted interviews and testing of blood and urine at baseline and at 12 and 15 weeks. The present study was performed at the Medical Corporation Seishinkai, Takara Clinic (Tokyo, Japan), and the Nerima Medical Association Minamimachi Clinic (Tokyo, Japan). All enrolled participants made written informed consent after being enlightened on the study procedures. 

### 2.4. Randomising and Blinding

All individuals were allocated by the allocation manager who assigned all participants once the target number of individuals had been reached. Subsequently, the allocation table was generated on the computer using random number generation and the complete method with factors of predefined variables as factors. The individuals were assigned either the DGLA-supplemented group or the placebo group at random according to the allocation table. The variables below were evenly distributed in both groups: age, sex, and JRQLQ score. The allocation table was given solely to the staff responsible for dispatching the test food, which was posted out in accordance with the allocation table. Data on randomization and allocation were kept confidential to the researchers, medical clinicians, research institution staff, ethics committee members, medical research centers, and participants until the time of the final analysis. The allocation table was sealed and kept by an independent controller pending retrieval with a key.

### 2.5. Treatment

Japanese cedar and cypress trees shed their pollen between February and April. Because PUFAs take 4–8 weeks after ingestion to be incorporated into the biomembrane [43,44], the intake of DGLA commenced approximately 8 weeks before the peak of pollen dispersal in March, starting on 8 January 2022. DGLA intake was continued until 23 April 2022, when pollen dispersal had mostly ceased, for a total of 15 weeks.

The participants took either 250 mg × 4 capsules of DGLA oil (1.0 g DGLA oil containing 314 mg DGLA) or a placebo containing 1.0 g of olive oil without DGLA daily during the study period of 15 weeks. DGLA oil was extracted from the biomass of submerged fermented *Mortierella alpina* and refined using high-purity processes [24]. The placebo and treatment capsules were identical in size, shape, and appearance. The participants were instructed not to change their daily habits, such as the quantity of alcohol consumed or exercise.

### 2.6. Primary Outcomes

#### 2.6.1. CSARS

Participants completed the CSARS daily, beginning one week before supplementation. They recorded nasal and eye symptoms, such as (1) sneezing, (2) runny nose, (3) blocked nose, (4) itchy eyes, and (5) watery eyes, on a 5-point scale from 0 (not at all) to 4 (very severe). Detailed questionnaire items are provided in Supplementary Data Table S1. The final score is the average of the scores for that week, with values calculated for each week from baseline to 15 weeks.

#### 2.6.2. JRQLQ

The participants received the JRQLQ at baseline and weeks 4, 6, 8, 10, 12, 14, and 15. Nasal and eye symptoms (1, sneezing; 2, runny nose; 3, blocked nose; 4, itchy nose; 5, itchy eyes; 6, watery eyes) were recorded on a 5-point scale from 0 (not at all) to 4 (very severe) following the time when symptoms were most severe 1–2 weeks before the recording date. Detailed questionnaire items are listed in Supplementary Data Table S2. The JRQLQ was evaluated using the total symptom score (TSS; total score of 1–6), nasal symptom score (TNSS; total score of 1–4), and ocular symptom score (TOSS; total score of 5 and 6).

### 2.7. Secondary Outcomes

Fasting serum polyunsaturated fatty acid (DGLA, AA, eicosapentaenoic acid (EPA), and docosahexaenoic acid (DHA)) composition were measured at baseline and week 15. Fasting serum nonspecific IgE, specific IgE (four species: house dust, mites, cedar, and cypress), fasting plasma interleukin-6 (IL-6), and histamine levels were measured at baseline and week 12. The participants completed nasal eosinophil counts from nasal secretions collected using cotton swabs at baseline and week 12. All measurements were performed by the LSI Medience Corporation (Tokyo, Japan).

### 2.8. Pollen Count

This study was conducted between 8 January 2022 and 23 April 2022. As the study sites (Takara Clinic and Minamimachi Clinic) are located in Tokyo, the pollen dispersal rate used was the average value for Tokyo from data obtained from the Tokyo Allergy Portal Site maintained by the Pollen Observation System of the Tokyo Metropolitan Bureau of Social Welfare and Public Health.

### 2.9. Dietary Assement

Participants took a dietary survey using the Calorie and Nutrition Diary [45] seven days before baseline and at weeks 12 and 15. The seven-day average of scores were taken to assess energy, protein, carbohydrate, fat, omega-6 fatty acid, and DGLA levels.

### 2.10. Safety Evaluation

Blood samples were collected at baseline, week 12, and week 15 under fasting conditions. Measurements of blood parameters included: white blood cell count; hemoglobin level; red blood cell count; platelet count; hematocrit; total protein; alanine aminotransferase; lactate dehydrogenase; aspartate aminotransferase; alkaline phosphatase; total bilirubin; albumin, γ-glutamyl transpeptidase; blood urea nitrogen; sodium; potassium; chlorine; LDL cholesterol; HDL cholesterol; triglyceride; total cholesterol; fasting plasma glucose; creatinine; and glycated hemoglobin. At baseline, week 12 and week 15, the following urine parameters were assessed: protein, glucose, and latent blood levels. The LSI Medience Corporation measured all plasma chemical variables. Systolic blood pressure and diastolic blood pressure, pulse rate, body weight and height, and body mass index measurements were taken at baseline, week 12, and week 15.

### 2.11. Sample Size

This study was considered a pre-trial and will be used as a reference for the calculation of cases in subsequent trials. In other studies examining nasal allergic reactions, the total number of participants was set to 19 [46], and the efficacy was verified. In this study, considering the subgroup formation, the target number of cases was 30. Considering the possibility of dropouts during the study period, three more participants per group were enrolled; thus, the number of cases was 36.

### 2.12. Statistical Analysis

Data in Tables are presented as the mean and standard deviation, and Figures are mean and standard error. Data were analyzed from the week just before pollen dispersal until week 15, when pollen dispersal was nearly over (the end of supplementation), using mixed models for repeated measures (MMRM) analysis. Participant backgrounds were compared between groups using the chi-square test or Student’s *t*-test. Blood parameters were compared between groups using Student’s *t*-test, and pre- and post-supplementation comparisons were made using the paired *t*-test. Nasal eosinophil count scores were compared between groups using the chi-square test. For all statistical analyses, two-sided tests were used, with a significance level of 5% (*p* < 0.05). We analyzed the data using the IBM SPSS statistical software (version 23 or higher; IBM Corp., Armonk, NY, USA).

## 3. Results

### 3.1. Participants

**Figure 1:**
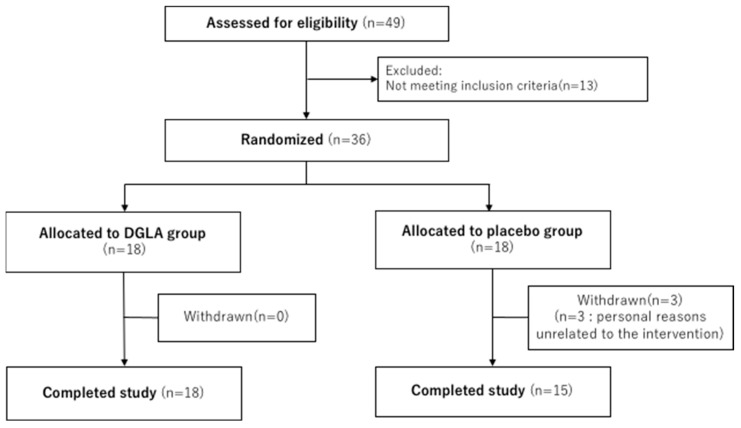
Flow diagram for the participants enrolled in the study.

As shown in Figure 1, 36 of the 49 potential participants met the inclusion criteria and were randomly assigned to one of the groups. The DGLA and placebo groups comprised 18 patients each. Three participants withdrew (withdrawal of agreements for personal reasons unrelated to the intervention), while the remaining participants completed the study. The background information of the subjects is presented in Table 1. One subject took medication for allergic rhinitis twice during the study, whereas the others did not.

In the DGLA group, after supplementation, the serum concentrations of DGLA were significantly higher than those in the placebo group (*p* = 0.011) and were significantly higher than the baseline scores at week 15 (*p* < 0.001) (Table 2). No items in the dietary survey differed significantly between the two groups (Table 3).

### 3.2. Pollen Counts

**Figure 2:**
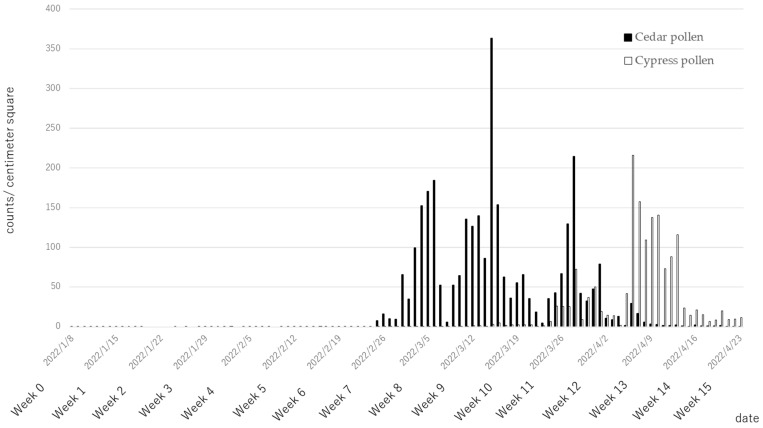
Cedar and cypress pollen counts per square centimeter in Tokyo, Japan, across the study period.

No cedar or cypress pollen were present at the start of supplementation. For cedar pollen, trees began to disperse pollen on 25 February 2022 (week 7), peaking in March (week 8–11) and continuing until late April (week 15). For cypress pollen, trees began to disperse pollen on 11 March 2022 (week 9), peaking in early April (weeks 13, 14) and continuing until late April (week 15) (Figure 2).

### 3.3. Primary Endpoint

#### 3.3.1. Cedar Pollen Associated Symptoms

**Figure 3:**
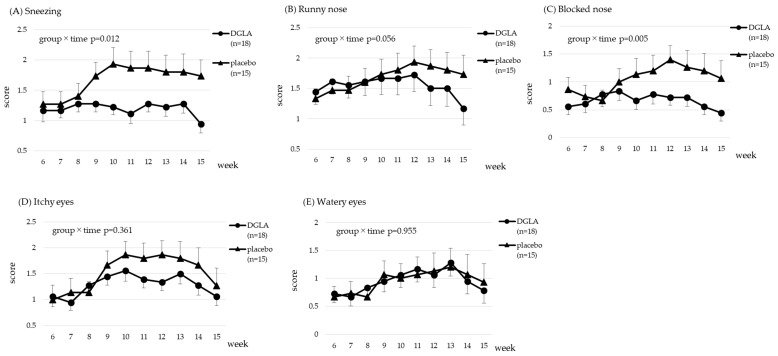
The CSARS score for cedar pollen in the DGLA and placebo groups.

**Figure 4:**
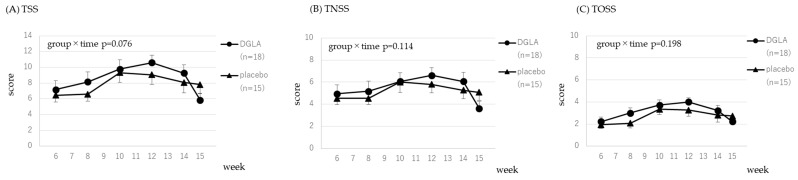
The JRQLQ score for cedar pollen in the DGLA and placebo groups.

The scores for the CSARS and the JRQLQ assessing symptoms related to cedar pollen are presented in Figure 3 and Figure 4. Data were analyzed from week 6, immediately before pollen dispersal, to week 15. All 33 subjects who completed the study had cedar-specific IgE, therefore, 33 subjects were included in the analysis. The time × group interaction effect was significant for sneezing and blocked nose; therefore, the DGLA group had significantly lower scores than the placebo group (*p* < 0.05, *p* < 0.01, respectively; Figure 3). Significant trends were observed in the reduction of the symptom of a runny nose in the CSARS and TSS in the JRQLQ (*p* = 0.056 and *p* = 0.076, respectively; Figure 3 and Figure 4). All scores are presented in Supplemental data Tables S3 and S4.

#### 3.3.2. Cypress Pollen Associated Symptoms

**Figure 5:**
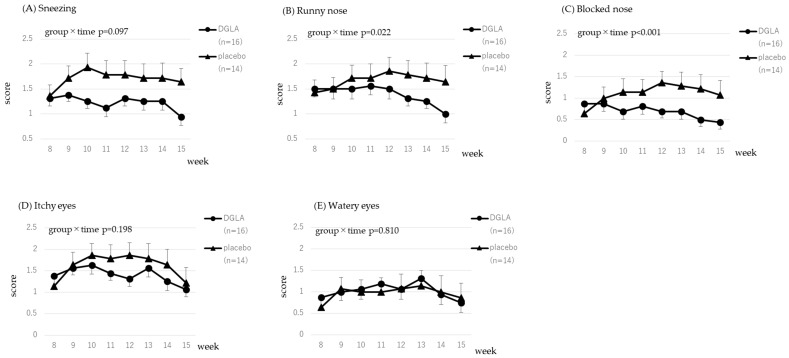
The CSARS score for cypress pollen in the DGLA and placebo groups.

**Figure 6:**
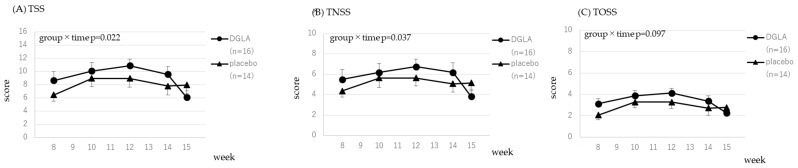
The JRQLQ score for cypress pollen in the DGLA and placebo treatment groups.

The scores for the CSARS and the JRQLQ related to cypress pollen are shown in Figure 5 and Figure 6. Data were analyzed from week 8, immediately before pollen dispersal, to week 15. Of the 33 subjects who completed the study, 30 had cypress-specific IgE and 30 were included in the analysis. The time × group interaction effect was significant for runny nose and blocked nose in the CSARS (*p* < 0.05, *p* < 0.01, respectively; Figure 5), and TSS and TNSS in the JRQLQ (both are *p* < 0.05; Figure 6); therefore, the DGLA group had significantly lower scores than the placebo group. Significant trends were observed in the reduction of the symptom of sneezing in the CSARS and TOSS in the JRQLQ (*p* = 0.097 and *p* = 0.097, respectively; Figure 5 and Figure 6). All scores are presented in Supplemental data Tables S5 and S6. 

### 3.4. Secondary Endpoint

The results of each blood parameter and nasal eosinophil count are shown in Table 4 and Table 5, respectively. No significant differences were found between the groups, but the specific IgE (each of the four species) and IL-6 scores were significantly higher than the baseline scores at week 12 (*p* < 0.001; Table 4). There were no significant differences in the nasal eosinophil counts between the two groups (Table 5). 

### 3.5. Adverse Events

Under the conditions of the study, six adverse events were identified in five participants during the study period. Two participants in the DGLA group experienced two events: inflammation of the paranasal sinuses and dysmenorrhea. Three participants (four events in the placebo group) reported headache, abdominal pain, coronavirus disease 2019 (COVID-19), and allergic rhinitis. The physician determined that all adverse events were unrelated to the intervention. Supplementary Table S7 shows the results of the blood and urine tests.

## 4. Discussion

To the best of our knowledge, this is the first study that evaluates the efficacy of DGLA in humans. Here, we confirm that 314 mg/day of DGLA is effective in improving nasal and eye allergy symptoms. Only three human studies on DGLA have been published to date; in one study published in 1977, 0.1–2 g of DGLA ethyl ester was administered as a single dose and the concentration of DGLA in the platelet membrane was determined [36]. In the other two studies, 50, 100, or 450 mg of DGLA triglycerides was administered for 4 weeks and the parameters in the blood and urine were checked to evaluate their safety [37,38]. The concentration of DGLA in the blood increased with the consumption of 50 mg or more of DGLA, with higher DGLA concentrations in the blood. The present study confirmed that 314 mg of DGLA intake increased the DGLA concentration in the blood and that the rate of increase was higher than in the 100 mg administration and lower than in the 450 mg administration in the previous studies [37,38].

The mechanism by which DGLA suppresses allergic symptoms is as follows: Allergens, such as pollen, bind to IgE, which further binds to mast cells, causing degranulation and the production of histamine, leukotriene, and thromboxane A2, leading to allergic symptoms in the nose and eyes. In nasal obstruction, leukotriene and thromboxane A2, which are eicosanoids metabolized from AA, are thought to increase vascular permeability and vasodilation. Vasodilation of the nose is thought to cause nasal blockage by narrowing the nostrils. On the other hand, histamine stimulates sensory nerves, causing sneezing and itchy eyes, and affects secretory glands, causing nasal secretions and running tears [4,32,33,34,47]. DGLA belongs to the same *n*-6 fatty acid family as AA and is thought to produce one series of PGs, unlike the two inflammation-inducing series of PGs produced by AA, which show anti-inflammatory effects [5,7,8,9,10,11,12,13,14,15,29,30,48]. DGLA produces metabolites that are antagonistic to the metabolism of these AAs, which are not as inflammatory as AAs and are consequently considered to suppress inflammation. A metabolite of DGLA, 15-HETrE, is also known to suppress pathways that produce inflammatory eicosanoids from AA [5,7,8,9,10,11,12,13,14,15,29,30,48]. These results suggest that DGLA exerts anti-allergic effects by inhibiting the production of inflammatory eicosanoids induced by AA. As DGLA improved sneezing symptoms in this study, it is also possible that DGLA inhibited histamine production. As shown earlier, DGLA suppresses the metabolism of leukotrienes by AA. Leukotriene B4 is a neutrophil migration factor that induces the expression of Fc receptors for IgE (FcεR) in B cells, enhances the effect of interleukin-4 (IL-4), and increases IgE production [49]. Therefore, DGLA may suppress sneezing by indirectly suppressing histamine release through leukotriene B4 production.

In this study, DGLA supplementation significantly improved nasal symptoms, such as sneezing, runny nose, and nasal obstruction. The mean change in itchy eyes in the CSARS was lower than that in the placebo group; however, the difference was not significant. There was no significant difference in tear fluid between the two groups. Considering the antiallergic mechanism of DGLA, it appears to be most effective against nasal congestion. There was an improvement in sneezing symptoms, possibly due to the indirect histamine-suppressive effect of leukotriene B4, but no significant difference in itchy eyes was observed between the two groups. The reason for this greater effect on sneezing may be that the inhibition of nasal vasodilation reduces the stimulation of sensory nerves. In addition, there was a significant improvement in nasal discharge, which affects the secretory glands, but no effect was noted on tear fluid, which affects the lacrimal glands, suggesting that the DGLA dosage used in this study had a slightly weaker effect on the secretory glands.

Regarding objective parameters, plasma-specific IgE, histamine, and IL-6 were found to increase at week 12, when pollen dispersal was higher, compared to the baseline. Although there was no difference in histamine levels between the groups in this study, the half-life of histamine released into the blood is very short (only a few minutes) [50], and most histamine in the blood is present in basophils with very little present in the plasma. The difference in the amount of histamine suppressed by DGLA would have been small given the mechanism, and it would have been difficult to detect a meaningful difference. Although there were no significant differences in blood IgE levels between the groups, DGLA supplementation did not significantly affect IgE levels, suggesting that the anti-allergic effect of DGLA was primarily due to its ability to inhibit the formation of degranulated substances by IgE.

In Figure 3, Figure 4, Figure 5 and Figure 6, CSARS and JRQLQ symptoms related to cedar and cypress pollen were evaluated. For all CSARS symptoms, the placebo group had higher symptoms from weeks 9 to 12, when pollen dispersal peaked, while the DGLA group showed little change in sneezing, nasal discharge, or nasal obstruction during this period, which was consistent with the effect of DGLA. In Supplemental data Figures S1 and S2, daily CSARS values and daily pollen counts are shown, and these also showed the same results as above. For JRQLQ, symptoms were higher in the placebo group from weeks 9 to 12, when pollen dispersal reached its peak, and then decreased. When all parameters comprising the TSS (1, sneezing; 2, runny nose; 3, blocked nose; 4, itchy nose; 5, itchy eyes; 6, watery eyes) were evaluated individually, it was found that, as in the CSCAR, more benefits were seen in nasal symptoms, with the greatest benefit seen in itchy nose (Supplemental data Figures S3 and S4). In the analysis of those with cypress pollen-specific IgE, we believed the reason for the observed effect in each score of the JRQLQ was the grater difference in symptoms between the placebo and DGLA groups at week 15, when cypress pollen levels are sharply reduced.

As for cedar IgE levels in the blood, week 12 was also the peak pollen dispersal period, and there was a significant increase compared with the baseline period of no pollen dispersal. For cypress, week 12 was just before the peak, but there was a significant increase compared to the baseline period when there was no pollen dispersal. These results confirmed that pollen affected the cedar pollen-specific IgE and cypress pollen-specific IgE levels in the blood during the consumption period, suggesting that DGLA affected nasal and eye allergic symptoms caused by cedar and cypress pollen.

In the analysis from the beginning to the end of supplementation, sneezing and nasal obstruction symptoms were significantly reduced in the DGLA group compared to that in the placebo group (Supplementary Data Tables S8 and S9). Although pollen was not present at the beginning of supplement administration, some nasal allergic symptoms were observed (sneezing: mean 1.07 ± 0.73, runny nose: mean 1.33 ± 0.90), suggesting that the subjects may have had allergic symptoms other than those caused by pollen. In addition, house dust- and mite-specific IgE in the blood significantly increased at week 12 compared to their respective levels before consumption (Table 4), suggesting that house dust and mites, which are present throughout the year, still affected the subjects during the study period. These findings suggest that the subjects were affected by house dust and mites during the study period and that DGLA may have improved nasal and eye allergy symptoms during this period, suggesting that DGLA may have improved allergic symptoms not only from pollen but also from house dust and mites.

Two subjective evaluations were conducted in this study: CSARS and JRQLQ. The CSARS was conducted daily and averaged over a week, while the JRQLQ was conducted once every two weeks, and the most severe symptoms were recorded. The CSARS is more specific (e.g., 2 for sneezing 6–10 times per day; Supplemental data Table S1), whereas the JRQLQ is less specific (no symptoms to very severe; Supplemental data Table S2); therefore, in this study, the CSARS was able to detect the effectiveness of DGLA more effectively. In other words, the question items and their content may have affected the detection of the effect.

In this study, we found that 314 mg/day of DGLA was effective against allergic symptoms, mainly in the nose. However, it is also necessary to examine the effects of IgE and histamine on the eyes. No previous studies have measured histamine production using DGLA, and inhibition of IgE production has been confirmed in animal studies [25]. Kawashima et al. [25] found that in NC/Nga mice, 0.6 g/kg of DGLA suppressed blood IgE levels compared to the control. Because the amount of DGLA used in the Kawashima et al. study [25], when converted to a human dose, is likely higher than the amount of DGLA used in this study, studies using higher doses of DGLA would be needed to determine the effects of DGLA on the eye.

## 5. Conclusions

This is the first study to confirm the effects of DGLA in human intervention study and the results of the present study suggest that DGLA is effective in reducing pollen-induced nasal and eye allergic symptoms. We believe that this study will help investigate the role of DGLA in the human body.

## Figures and Tables

**Table 1:** Baseline information for DGLA and placebo treatment groups.

Variable	DGLA Group (n = 18)	Placebo Group (n = 15)	*p* Value
n (Men/Women)	7/11	4/11	0.712
Age (years)	48.1 ± 11.6	49.7 ± 13.0	0.698
Weight (kg)	59.3 ± 14.5	61.3 ± 24.5	0.772
Height (cm)	163.6 ± 9.8	160.3 ± 7.1	0.290
BMI (kg/m^2^)	21.9 ± 3.4	23.4 ± 7.2	0.438

Abbreviations: BMI, body mass index; DGLA, dihomo-gamma-linoleic acid.

**Table 2:** Plasma dihomo-gamma-linoleic acid, arachidonic acid, eicosapentaenoic acid, and docosahexaenoic acid concentrations in the DGLA and placebo treatment groups.

Variable	Baseline			Week 15		
	DGLA Group (n = 18)	Placebo Group (n = 15)	*p* Value	DGLA Group (n = 18)	Placebo Group (n = 15)	*p* Value
DGLA (µg/mL)	42.9 ± 20.9	44.9 ± 18.5	0.776	62.1 ± 19.5 *$$$	49.3 ± 15.7	0.011
AA (µg/mL)	211.8 ± 64.4	201.1 ± 46.7	0.593	228.3 ± 59.6	200.1 ± 30.7	0.106
EPA (µg/mL)	47.1 ± 28.6	47.8 ± 36.8	0.948	40.0 ± 24.3	59.0 ± 56.8	0.118
DHA (µg/mL)	104.8 ± 38.1	106.0 ± 37.3	0.930	107.7 ± 52.5	115.1 ± 34.1	0.500

Abbreviations: DGLA, dihomo-gamma-linoleic acid; AA, arachidonic acid; EPA, eicosapentaenoic acid; DHA, docosahexaenoic acid. Group differences were assessed using the Student’s *t*-test; significance: * *p* < 0.05. Between baseline and week 12, differences were assessed using a paired *t*-test; $$$ *p* < 0.001.

**Table 3:** Dietary survey for DGLA and placebo treatment groups.

Variable	Time	DGLA Group (n = 18)	Placebo Group (n = 15)	*p* Value
Energy (kcal)	Baseline	2345.9 ± 837.2	2309.2 ± 433.0	0.872
	Week 12	2725.1 ± 1201.0	2568.8 ± 727.9	0.649
	Week 15	2773.7 ± 1643.3	2778.0 ± 760.7	0.992
Protein (g)	Baseline	95.3 ± 46.9	90.7 ± 17.6	0.702
	Week 12	108.3 ± 49.3	101.2 ± 31.8	0.624
	Week 15	105.4 ± 48.6	110.5 ± 32.1	0.722
Fat (g)	Baseline	85.5 ± 43.2	78.9 ± 19.5	0.564
	Week 12	95.6 ± 49.0	89.6 ± 29.4	0.667
	Week 15	96.7 ± 49.7	101.1 ± 34.1	0.768
Carbonate (g)	Baseline	284.5 ± 82.9	294.6 ± 63.5	0.696
	Week 12	342.8 ± 169.1	326.6 ± 89.1	0.727
	Week 15	358.2 ± 269.1	341.3 ± 96.6	0.806
Omega-6 fatty acid (g)	Baseline	14.4 ± 9.0	14.0 ± 3.8	0.875
	Week 12	16.3 ± 8.7	15.3 ± 6.7	0.724
	Week 15	16.8 ± 7.2	18.3 ± 7.4	0.562
DGLA (mg)	Baseline	55.6 ± 29.7	47.9 ± 13.3	0.333
	Week 12	60.9 ± 27.9	56.7 ± 22.8	0.636
	Week 15	59.7 ± 29.8	61.1 ± 20.4	0.874

Abbreviations: DGLA, dihomo-gamma-linoleic acid.

**Table 4:** Blood scores for the DGLA and placebo treatment groups.

Variable	Baseline			Week 12		
	DGLA Group (n = 18)	Placebo Group (n = 15)	*p* Value	DGLA Group (n = 18)	Placebo Group (n = 15)	*p* Value
Cedar-specific IgE (UA/mL)	10.8 ± 15.9	14.3 ± 19.5	0.579	14.6 ± 20.2 $$$	16.1 ± 24.1 $$$	0.850
Cypress-specific IgE (UA/mL)	1.3 ± 1.6	2.8 ± 3.2	0.097	1.8 ± 2.5 $$$	3.2 ± 3.9 $$$	0.235
House dust-specific IgE (UA/mL)	2.7 ± 4.1	8.6 ± 25.5	0.343	2.7 ± 4.2 $$$	8.1 ± 24.3 $$$	0.345
Mite-specific IgE (UA/mL)	2.9 ± 4.3	8.5 ± 25.5	0.361	3.4 ± 5.3 $$$	8.8 ± 25.4 $$$	0.385
Nonspecific IgE (IU/mL)	158.5 ± 183.3	140.1 ± 214.6	0.792	176.6 ± 188.6	146.3 ± 235.5	0.583
Histamine (ng/mL)	1.1 ± 0.9	0.8 ± 0.8	0.347	1.4 ± 1.2	1.4 ± 1.2	0.876
IL-6 (pg/mL)	1.3 ± 0.7	1.4 ± 1.3	0.639	1.5 ± 0.9 $$$	1.3 ± 0.8 $$$	0.373

Abbreviations: DGLA, dihomo-gamma-linoleic acid. Between baseline and week 12, differences were assessed using a paired *t*-test; $$$ *p* < 0.001.

**Table 5:** Nasal score for the DGLA and placebo treatment groups.

Variable	Baseline			Week 12		
	DGLA Group (n = 18)	Placebo Group (n = 15)	*p* Value	DGLA Group (n = 18)	Placebo Group (n = 15)	*p* Value
Nasal eosinophil (−)	15	12	1.000	13	11	1.000
Nasal eosinophil (±)	0	0	N.A.	0	0	N.A.
Nasal eosinophil (+)	2	3	0.639	2	1	1.000
Nasal eosinophil (2+)	0	0	N.A.	3	3	1.000
Nasal eosinophil (3+)	1	0	1.000	0	0	N.A.

Abbreviation: DGLA, dihomo-gamma-linoleic acid.

## Data Availability

The data supporting the results of this study are available from the corresponding author when reasonably requested.

## Supplementary Materials

The following supporting information can be downloaded from: https://www.mdpi.com/article/10.3390/nu15153465/s1, Table S1: CSARS; Table S2: JRQLQ; Table S3: The CSARS score for cedar pollen in the DGLA and placebo groups; Table S4: The JRQLQ score for cedar pollen in the DGLA and placebo treatment groups; Table S5: The CSARS score for cypress pollen in the DGLA and placebo groups; Table S6: The JRQLQ score for cypress pollen in the DGLA and placebo treatment groups; Table S7: Safety evaluation results at baseline, Week 12, and Week 15; Table S8: Score of CSARS for the DGLA and placebo treatment groups; Table S9: Score of JRQLQ for the DGLA and placebo treatment groups; Figure S1: The daily of CSARS score for cedar pollen in the DGLA and placebo groups; Figure S2: The daily CSARS score for cypress pollen in the DGLA and placebo groups; Figure S3: The all parameters of JRQLQ score for cedar pollen in the DGLA and placebo groups; Figure S4: The all parameters of JRQLQ score for cypress pollen in the DGLA and placebo groups.
