# Supplementary material for: Supplementation of Dihomo-γ-Linolenic Acid for Pollen-Induced Allergic Symptoms in Healthy Subjects: A Randomized, Double-Blinded, Placebo-Controlled Trial
Source: Nutrients. 2023 Aug 5;15(15):3465. doi: 10.3390/nu15153465 (PMC10421109; doi:10.3390/nu15153465)
Supplement: Supplementary file 1 [file nutrients-15-03465-s001.zip › nutrients-2518435-supplementary.pdf]

Supplemental Tables

Supplemental data Table S1. CSARS.

	Score				
**Variable**	**0**	**1**	**2**	**3**	**4**
Sneezing 1	0	1 to 5	6 to 10	11 to 20	>21
Runny nose 2	0	1 to 5	6 to 10	11 to 20	>21
Blocked nose	None	Nasal blockage without oral breathing	Severe nasal blockage causing occasional oral breathing in a day	Severe nasal blockage causing prolonged oral breathing in a day	Completely obstructed all day
Itchy eyes	None	Not enough to rub one's eyes	Occasionally rubbing one's eyes	Frequently rubbing one's eyes	≥ 3
Watery eyes	None	Not enough to wipe tears	Occasionally wiping tears	Frequently wiping tears	≥ 3

[1] Average number of episodes of sneezing in a day
[2] Average number of episodes of nose blowing a day

Supplemental data Table S2. JRQLQ.

	Score				
**Variable**	**0**	**1**	**2**	**3**	**4**
Sneezing	None	Mild	Moderate	Severe	Extremely Severe
Runny nose	None	Mild	Moderate	Severe	Extremely Severe
Blocked nose	None	Mild	Moderate	Severe	Extremely Severe
Itchy nose	None	Mild	Moderate	Severe	Extremely Severe
Itchy eyes	None	Mild	Moderate	Severe	Extremely Severe
Watery eyes	None	Mild	Moderate	Severe	Extremely Severe

Supplemental data Table S3. The CSARS score for cedar pollen in the DGLA and placebo groups.

Variable	Group	Week 6	Week 7	Week 8	Week 9	Week 10	Week 11	Week 12	Week 13	Week 14	Week 15	p-value		
												Group	Time	Group* Time
sneezing	DGLA group (n=18)	1.17 ± 0.79	1.17 ± 0.51	1.28 ± 0.57	1.28 ± 0.57	1.22 ± 0.57	1.11 ± 0.68	1.28 ± 0.57	1.22 ± 0.65	1.28 ± 0.67	0.94 ± 0.64	0.046*	0.007**	0.012*
	Placebo group (n=15)	1.27 ± 0.80	1.27 ± 0.80	1.40 ± 0.83	1.73 ± 0.88	1.93 ± 1.03	1.87 ± 1.06	1.87 ± 1.06	1.80 ± 1.08	1.80 ± 1.15	1.73 ± 1.03			
Runny nose	DGLA group (n=18)	1.44 ± 0.92	1.61 ± 0.92	1.56 ± 0.70	1.61 ± 0.85	1.67 ± 0.97	1.67 ± 0.77	1.72 ± 1.02	1.50 ± 0.86	1.50 ± 0.92	1.17 ± 0.99	0.654	0.007**	0.056#
	Placebo group (n=15)	1.33 ± 0.62	1.47 ± 0.74	1.47 ± 0.92	1.60 ± 0.91	1.73 ± 0.96	1.80 ± 1.08	1.93 ± 1.03	1.87 ± 1.06	1.80 ± 1.15	1.73 ± 1.22			
Blocked nose	DGLA group (n=18)	0.56 ± 0.62	0.61 ± 0.70	0.78 ± 0.94	0.83 ± 0.71	0.67 ± 0.78	0.78 ± 0.73	0.72 ± 0.57	0.72 ± 0.67	0.56 ± 0.62	0.44 ± 0.62	0.146	0.002**	0.005**
	Placebo group (n=15)	0.87 ± 0.83	0.73 ± 0.80	0.67 ± 0.72	1.00 ± 0.93	1.13 ± 1.13	1.20 ± 1.08	1.40 ± 0.99	1.27 ± 1.16	1.20 ± 1.21	1.07 ± 1.22			
Itchy eyes	DGLA group (n=18)	1.06 ± 0.80	0.94 ± 0.64	1.28 ± 0.89	1.44 ± 0.70	1.56 ± 0.86	1.39 ± 0.70	1.33 ± 0.69	1.50 ± 0.86	1.28 ± 0.83	1.06 ± 0.73	0.392	0.000**	0.361
	Placebo group (n=15)	1.00 ± 1.07	1.13 ± 1.06	1.13 ± 0.83	1.67 ± 1.05	1.87 ± 0.99	1.80 ± 1.15	1.87 ± 1.06	1.80 ± 1.26	1.67 ± 1.29	1.27 ± 1.33			
Watery eyes	DGLA group (n=18)	0.72 ± 0.67	0.67 ± 0.69	0.83 ± 0.92	0.94 ± 0.80	1.06 ± 0.94	1.17 ± 0.99	1.06 ± 0.94	1.28 ± 1.02	0.94 ± 0.94	0.78 ± 0.94	0.976	0.000**	0.955
	Placebo group (n=15)	0.67 ± 0.72	0.73 ± 0.80	0.67 ± 0.62	1.07 ± 0.96	1.00 ± 1.00	1.07 ± 1.22	1.13 ± 1.25	1.20 ± 1.32	1.07 ± 1.39	0.93 ± 1.28			

Abbreviation: DGLA, dihomo-gamma-linoleic acid. **$p<0.01$, *$p < 0.05$, #$p < 0.1$.

Supplemental data Table S4. The JRQLQ score for cedar pollen in the DGLA and placebo treatment groups.

Variable	Group	Week 6	Week 8	Week 10	Week 12	Week 14	Week 15	p-value		
								Group	Time	Group* Time
Total Symptom Score (TSS)	DGLA group (n=18)	7.17 ± 4.89	8.17 ± 5.40	9.78 ± 4.95	10.6 ± 3.88	9.28 ± 4.35	5.83 ± 3.79	0.662	0.000**	0.076#
	Placebo group (n=15)	6.47 ± 3.44	6.60 ± 3.44	9.33 ± 4.79	9.07 ± 4.83	8.07 ± 5.04	7.80 ± 4.51			
Total Nasal Symptom Score (TNSS)	DGLA group (n=18)	4.94 ± 3.44	5.17 ± 3.88	6.06 ± 3.42	6.61 ± 2.87	6.06 ± 3.51	3.61 ± 2.77	0.825	0.000**	0.114
	Placebo group (n=15)	4.53 ± 2.26	4.53 ± 2.26	6.00 ± 3.68	5.80 ± 2.96	5.27 ± 2.99	5.07 ± 2.84			
Total Ocular Symptom Score (TOSS)	DGLA group (n=18)	2.22 ± 1.70	3.00 ± 1.91	3.72 ± 2.02	4.00 ± 1.64	3.22 ± 1.93	2.22 ± 1.59	0.484	0.000**	0.198
	Placebo group (n=15)	1.93 ± 1.33	2.07 ± 1.58	3.33 ± 1.88	3.27 ± 2.19	2.80 ± 2.40	2.73 ± 1.91			

Abbreviation: DGLA, dihomo-gamma-linoleic acid. **$p<0.01$, #$p < 0.1$.

Supplemental data Table S5. The CSARS score for cypress pollen in the DGLA and placebo groups.

Variable	Group	Week 8	Week 9	Week 10	Week 11	Week 12	Week 13	Week 14	Week 15	p-value		
										Group	Time	Group* Time
sneezing	DGLA group (n=16)	1.31 ± 0.60	1.38 ± 0.50	1.25 ± 0.58	1.13 ± 0.72	1.31 ± 0.60	1.25 ± 0.68	1.25 ± 0.68	0.94 ± 0.68	0.082#	0.129	0.097#
	Placebo group (n=14)	1.36 ± 0.84	1.71 ± 0.91	1.93 ± 1.07	1.79 ± 1.05	1.79 ± 1.05	1.71 ± 1.07	1.71 ± 1.14	1.64 ± 1.01			
Runny nose	DGLA group (n=16)	1.50 ± 0.63	1.50 ± 0.82	1.50 ± 0.82	1.56 ± 0.73	1.50 ± 0.82	1.31 ± 0.60	1.25 ± 0.58	1.00 ± 0.73	0.339	0.053#	0.022*
	Placebo group (n=14)	1.43 ± 0.94	1.50 ± 0.85	1.71 ± 0.99	1.71 ± 1.07	1.86 ± 1.03	1.79 ± 1.05	1.71 ± 1.14	1.64 ± 1.22			
Blocked nose	DGLA group (n=16)	0.88 ± 0.96	0.88 ± 0.72	0.69 ± 0.70	0.81 ± 0.75	0.69 ± 0.60	0.69 ± 0.70	0.50 ± 0.63	0.44 ± 0.63	0.185	0.092#	0.000**
	Placebo group (n=14)	0.64 ± 0.74	1.00 ± 0.96	1.14 ± 1.17	1.14 ± 1.10	1.36 ± 1.01	1.29 ± 1.20	1.21 ± 1.25	1.07 ± 1.27			
Itchy eyes	DGLA group (n=16)	1.38 ± 0.89	1.56 ± 0.63	1.63 ± 0.81	1.44 ± 0.63	1.31 ± 0.70	1.56 ± 0.81	1.25 ± 0.86	1.06 ± 0.68	0.481	0.000**	0.198
	Placebo group (n=14)	1.14 ± 0.86	1.64 ± 1.08	1.86 ± 1.03	1.79 ± 1.19	1.86 ± 1.10	1.79 ± 1.31	1.64 ± 1.34	1.21 ± 1.37			
Watery eyes	DGLA group (n=16)	0.88 ± 0.96	1.00 ± 0.82	1.06 ± 0.93	1.19 ± 0.98	1.06 ± 0.93	1.31 ± 1.01	0.94 ± 0.93	0.75 ± 0.93	0.886	0.007**	0.810
	Placebo group (n=14)	0.64 ± 0.63	1.07 ± 1.00	1.00 ± 1.04	1.00 ± 1.24	1.07 ± 1.27	1.14 ± 1.35	1.00 ± 1.41	0.86 ± 1.29			

Abbreviation: DGLA, dihomo-gamma-linoleic acid. **p<0.01, *p < 0.05, #p < 0.1.

Supplemental data Table S6. The JRQLQ score for cypress pollen in the DGLA and placebo treatment groups.

Variable	Group	Week 8	Week 10	Week 12	Week 14	Week 15	p-value		
							Group	Time	Group*Time
Total Symptom Score (TSS)	DGLA group (n=16)	8.63 ± 5.43	10.06 ± 5.20	10.88 ± 4.01	9.56 ± 4.35	6.06 ± 3.91	0.484	0.000**	0.022*
	Placebo group (n=14)	6.43 ± 3.50	8.93 ± 4.70	8.93 ± 4.98	7.79 ± 5.10	7.93 ± 4.65			
Total Nasal Symptom Score (TNSS)	DGLA group (n=16)	5.50 ± 3.97	6.19 ± 3.60	6.75 ± 3.02	6.19 ± 3.71	3.81 ± 2.88	0.621	0.001**	0.037*
	Placebo group (n=14)	4.36 ± 2.24	5.64 ± 3.54	5.64 ± 3.00	5.07 ± 3.00	5.14 ± 2.93			
Total Ocular Symptom Score (TOSS)	DGLA group (n=16)	3.13 ± 1.86	3.88 ± 2.03	4.13 ± 1.67	3.38 ± 1.96	2.25 ± 1.53	0.389	0.000**	0.094#
	Placebo group (n=14)	2.07 ± 1.64	3.29 ± 1.94	3.29 ± 2.27	2.71 ± 2.46	2.79 ± 1.97			

Abbreviation: DGLA, dihomo-gamma-linoleic acid. **$p<0.01$, *$p < 0.05$, #$p < 0.1$.

Supplemental data Table S7. Safety evaluation results at Baseline, Week 12, and Week 15.

**Variable**	**Time**	**DGLA group (n=18)**	**Placebo group (n=15)**	**p value**
SBP (mmHg)	Baseline	121.9±14.3	119.5±14.9	0.639
	Week 12	118.1±11.9	118.7±11.8	0.884
	Week 15	113.4±10.9	115.8±15.2	0.620
DBP (mmHg)	Baseline	80.2±10.7	81.3±11.6	0.803
	Week 12	75.9±10.2	79.9±11.6	0.316
	Week 15	74.4±9.1	77.1±12.4	0.484
Pulse rate (bpm)	Baseline	70.9±8.8	74.6±12.9	0.354
	Week 12	71.5±10.6	72.2±11.5	0.858
	Week 15	72.3±10.0	75.1±11.8	0.486
WBC (μL)	Baseline	5327.8±1083.5	5046.7±1731.6	0.590
	Week 12	5455.6±1216.2	4960.0±1504.7	0.314
	Week 15	5294.4±1041.8	4920.0±1186.4	0.348
RBC ($\times 10^4$/μL)	Baseline	460.3±35.3	457.1±45.7	0.826
	Week 12	462.9±35.6	457.9±50.6	0.746
	Week 15	451.7±32.8	444.6±41.6	0.598
Hb (g/dL)	Baseline	13.8±1.3	13.9±0.9	0.934
	Week 12	13.8±1.3	13.8±1.1	0.954
	Week 15	13.5±1.2	13.5±1.0	0.982
Ht (%)	Baseline	43.6±3.5	43.2±3.2	0.757
	Week 12	43.6±3.5	43.0±3.7	0.655

	Week 15	42.9±3.3	42.5±3.3	0.728
Platelet count (10^4/μL)	Baseline	27.1±6.1	24.3±4.9	0.151
	Week 12	27.5±5.9	25.9±6.1	0.454
	Week 15	26.5±5.9	23.9±4.8	0.182
AST (U/L)	Baseline	20.2±6.0	20.9±4.6	0.703
	Week 12	20.3±7.0	20.9±4.7	0.771
	Week 15	20.9±7.2	20.3±6.6	0.818
ALT (U/L)	Baseline	17.3±9.5	17.9±7.5	0.840
	Week 12	18.7±12.1	18.1±6.7	0.874
	Week 15	20.6±16.1	17.7±7.2	0.492
γGTP (U/L)	Baseline	30.1±32.1	20.2±9.4	0.227
	Week 12	31.5±31.2	22.8±11.7	0.285
	Week 15	34.0±47.0	21.2±10.2	0.271
ALP (U/L)	Baseline	69.2±18.4	70.3±22.9	0.881
	Week 12	74.3±18.9	73.6±22.2	0.926
	Week 15	71.3±21.9	69.4±21.1	0.804
LDH (U/L)	Baseline	202.0±26.7	199.0±31.2	0.771
	Week 12	201.4±28.3	193.8±27.2	0.439
	Week 15	198.0±24.5	191.1±34.9	0.527
Total bilirubin (mg/dL)	Baseline	0.8±0.3	0.7±0.3	0.567
	Week 12	0.7±0.3	0.8±0.2	0.428
	Week 15	0.8±0.3	0.8±0.3	0.886
Total protein (g/dL)	Baseline	7.2±0.3	7.4±0.4	0.283

	Week 12	7.2±0.4	7.2±0.5	0.922
	Week 15	7.1±0.3	7.1±0.3	0.592
BUN (mg/dL)	Baseline	12.4±3.5	13.3±3.8	0.468
	Week 12	12.8±2.4	14.7±2.5	0.039
	Week 15	11.3±2.9	13.6±2.8	0.027
Creatinine (mg/dL)	Baseline	0.7±0.2	0.7±0.1	0.986
	Week 12	0.7±0.1	0.7±0.1	0.729
	Week 15	0.7±0.2	0.7±0.1	0.985
Na (mEq/L)	Baseline	140.1±2.1	140.6±2.0	0.498
	Week 12	140.8±1.8	141.2±1.9	0.525
	Week 15	141.1±1.1	141.9±2.2	0.169
K (mEq/L)	Baseline	4.3±0.3	4.5±1.0	0.461
	Week 12	4.3±0.3	4.3±0.4	0.887
	Week 15	4.0±0.2	3.9±0.3	0.197
Cl (mEq/L)	Baseline	100.9±2.3	101.0±1.7	0.937
	Week 12	101.3±2.0	101.9±2.6	0.525
	Week 15	101.7±1.7	101.9±1.4	0.695
Total cholesterol (mg/dL)	Baseline	227.6±38.1	231.8±34.5	0.743
	Week 12	233.8±43.8	233.0±31.8	0.954
	Week 15	233.8±269.1	232.3±29.9	0.907
HDL-cholesterol (mg/dL)	Baseline	72.8±14.2	77.7±22.7	0.476
	Week 12	75.5±15.9	79.6±21.1	0.540
	Week 15	76.8±16.4	81.4±19.4	0.471

LDL-cholesterol (mg/dL)	Baseline	130.8±32.2	128.9±24.2	0.843
	Week 12	137.8±39.8	130.8±26.7	0.550
	Week 15	135.0±37.4	126.9±28.1	0.485
TG (mg/dL)	Baseline	90.3±61.2	88.3±41.5	0.912
	Week 12	86.3±41.8	89.4±67.5	0.877
	Week 15	95.7±45.3	84.5±52.5	0.524
PG (mg/dL)	Baseline	83.6±8.1	88.3±8.0	0.103
	Week 12	86.3±11.7	89.5±15.6	0.527
	Week 15	85.7±7.8	87.6±9.4	0.531
HbA1c (NGSP)	Baseline	5.4±0.3	5.4±0.3	0.954
	Week 12	5.5±0.3	5.5±0.3	0.835
	Week 15	5.5±0.3	5.5±0.3	0.960

Data are represented as mean ± SD; body mass index (BMI), systolic blood pressure (SBP), diastolic blood pressure (DBP), white blood cell (WBC) count, red blood cell (RBC) count, hemoglobin (Hb), hematocrit (Ht), aspartate aminotransferase (AST), alanine aminotransferase (ALT), lactate dehydrogenase (LDH), alkaline phosphatase (ALP), γ-glutamyl trans-peptidase (γGTP), blood urea nitrogen (BUN), sodium (Na), chlorine (Cl), potassium (K), triglyceride (TG), fasting plasma glucose concentration (FPG), and glycated hemoglobin (HbA1c).

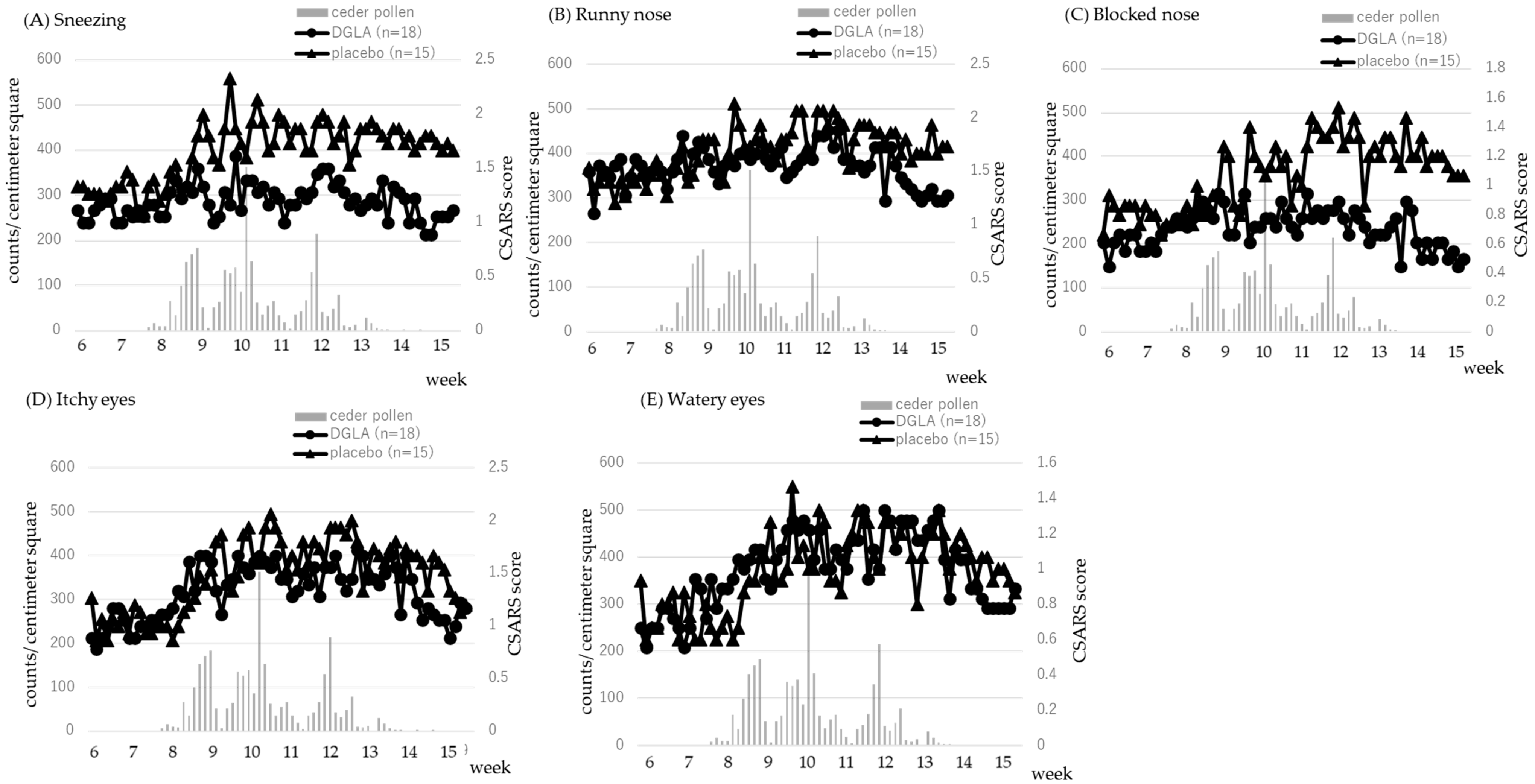

Supplemental data Figure S1. The daily of CSARS score for cedar pollen in the DGLA and placebo groups.

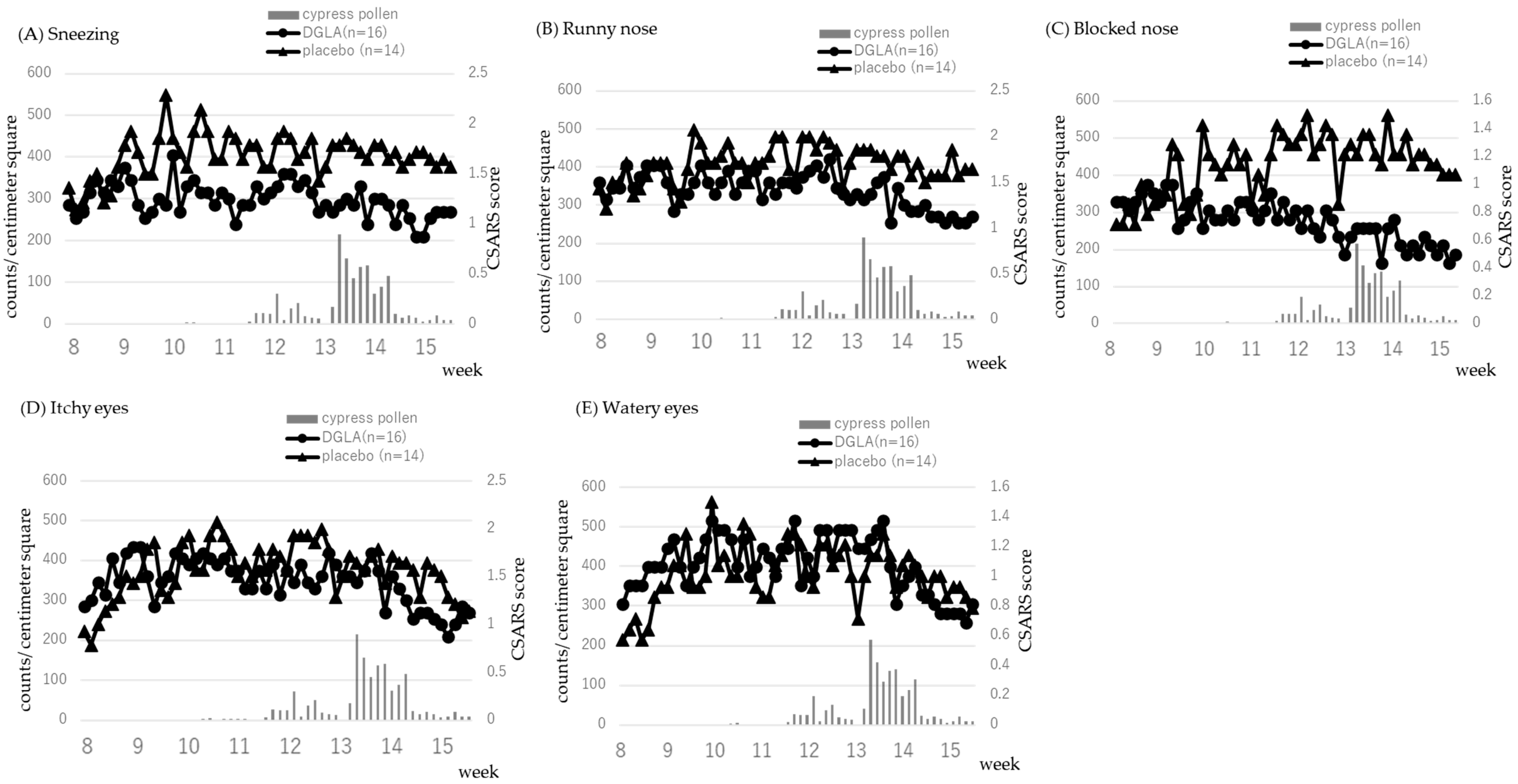

Supplemental data Figure S2. The daily of CSARS score for cypress pollen in the DGLA and placebo groups.

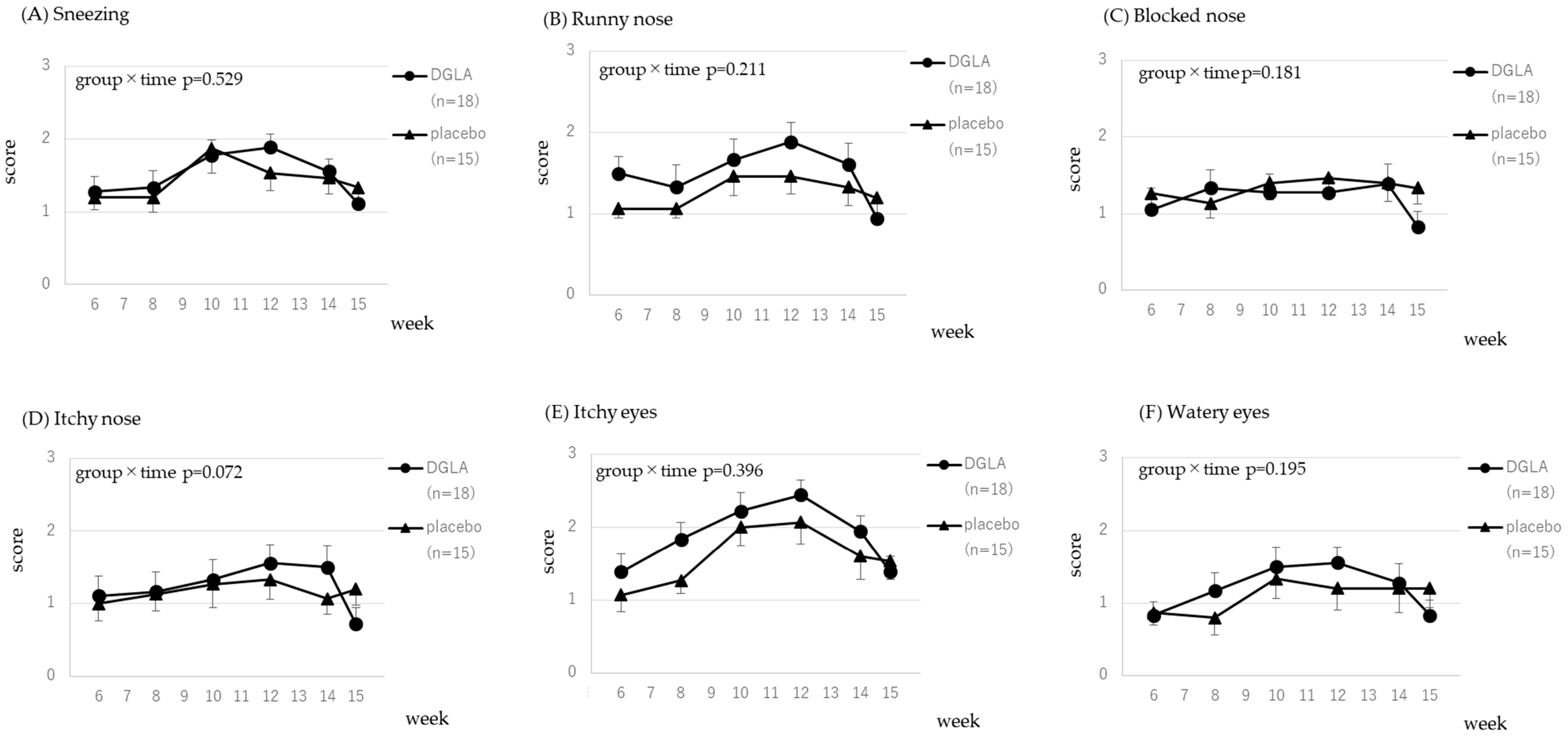

Supplemental data Figure S3. The all parameters of JRQLQ score for cedar pollen in the DGLA and placebo groups.

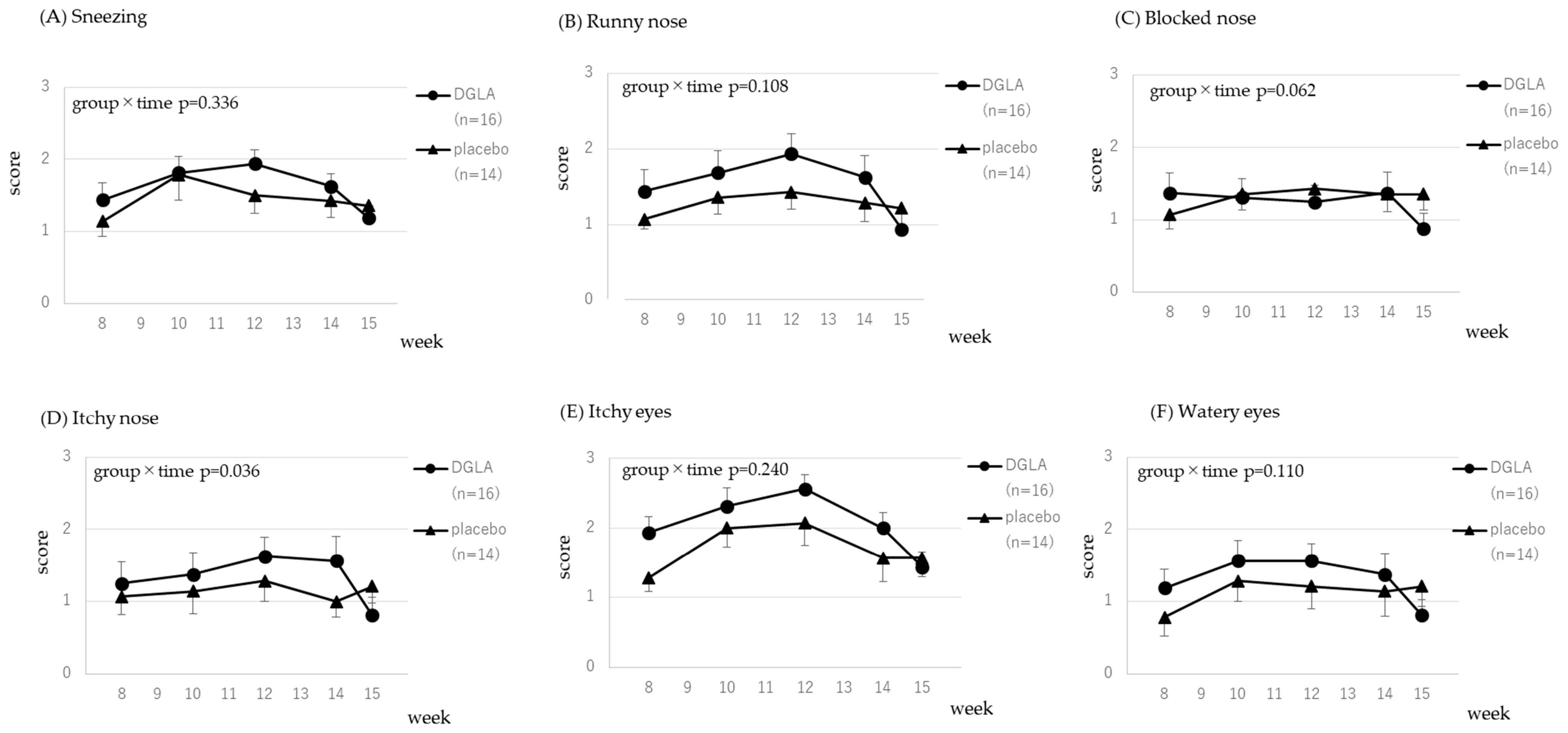

Supplemental data Figure S4. The all parameters of JRQLQ score for cypress pollen in the DGLA and placebo groups.

Supplemental data Table S8. Score of CSARS for the DGLA and placebo treatment groups.

Variable	Group	Week 0	Week 1	Week 2	Week 3	Week 4	Week 5	Week 6	Week 7	Week 8	Week 9	Week 10	Week 11	Week 12	Week 13	Week 14	Week 15	p-value		
																		Group	Time	Group* Time
sneezing	DGLA group (n=18)	0.94±0.54	1.00±0.49	1.06±0.73	0.94±0.64	1.00±0.59	1.11±0.68	1.17±0.79	1.17±0.51	1.28±0.57	1.28±0.57	1.22±0.55	1.11±0.68	1.28±0.57	1.22±0.65	1.28±0.67	0.94±0.64	0.115	0.000**	0.001*
	Placebo group (n=15)	1.07±0.70	1.00±0.85	1.13±0.74	0.93±0.70	1.20±0.94	1.20±0.77	1.27±0.80	1.27±0.80	1.40±0.83	1.73±0.88	1.93±1.03	1.87±1.06	1.87±1.06	1.80±1.08	1.80±1.15	1.73±1.03			
Runny nose	DGLA group (n=18)	1.17±0.86	1.39±0.92	1.44±0.92	1.50±0.86	1.33±0.77	1.50±0.62	1.44±0.92	1.61±0.92	1.56±0.70	1.61±0.85	1.67±0.97	1.67±0.77	1.72±1.02	1.50±0.86	1.50±0.92	1.17±0.99	0.804	0.000**	0.124
	Placebo group (n=15)	1.33±0.90	1.33±0.90	1.40±0.74	1.20±0.56	1.47±0.92	1.33±0.82	1.33±0.62	1.47±0.74	1.47±0.92	1.60±0.91	1.73±0.96	1.80±1.08	1.93±1.03	1.87±1.06	1.80±1.15	1.73±1.22			
Blocked nose	DGLA group (n=18)	0.67±0.91	0.61±0.78	0.61±0.61	0.39±0.50	0.44±0.51	0.56±0.62	0.56±0.62	0.61±0.70	0.78±0.94	0.83±0.71	0.67±0.78	0.78±0.73	0.72±0.57	0.72±0.67	0.56±0.62	0.44±0.62	0.199	0.000**	0.013*
	Placebo group (n=15)	0.47±0.52	0.60±0.74	0.80±0.77	0.60±0.74	0.67±0.82	0.73±0.70	0.87±0.83	0.73±0.80	0.67±0.72	1.00±0.93	1.13±1.13	1.20±1.08	1.40±0.99	1.27±1.16	1.20±1.21	1.07±1.22			
Itchy eyes	DGLA group (n=18)	1.22±0.94	0.94±0.87	0.89±0.68	0.89±0.68	0.83±0.62	0.94±0.80	1.06±0.80	0.94±0.64	1.28±0.89	1.44±0.70	1.56±0.86	1.39±0.70	1.33±0.69	1.50±0.86	1.28±0.83	1.06±0.73	0.550	0.000**	0.383
	Placebo group (n=15)	1.00±0.76	0.93±0.80	0.87±0.74	0.80±0.86	1.00±1.00	1.07±0.96	1.00±1.07	1.13±1.06	1.13±0.83	1.67±1.05	1.87±0.99	1.80±1.15	1.87±1.06	1.80±1.26	1.67±1.29	1.27±1.33			
Watery eyes	DGLA group (n=18)	0.50±0.71	0.61±0.61	0.61±0.61	0.50±0.71	0.56±0.62	0.83±0.79	0.72±0.67	0.67±0.69	0.83±0.92	0.94±0.80	1.06±0.94	1.17±0.99	1.06±0.94	1.28±1.02	0.94±0.94	0.78±0.94	0.410	0.000**	0.309
	Placebo group (n=15)	0.67±0.72	0.60±0.74	0.60±0.63	0.53±0.83	0.60±0.74	0.67±0.82	0.67±0.72	0.73±0.80	0.67±0.62	1.07±0.96	1.00±1.00	1.07±1.22	1.13±1.25	1.20±1.32	1.07±1.39	0.93±1.28			

**$p < 0.01$, *$p < 0.05$

Supplemental data Table S9. Score of JRQLQ for the DGLA and placebo treatment groups.

Variable	Group	Week 0	Week 4	Week 6	Week 8	Week 10	Week 12	Week 14	Week 15	p-value		
										Group	Time	Group* Time
Total Symptom Score (TSS)	DGLA group (n=18)	7.39±5.30	7.28±5.18	7.17±4.89	8.17±5.40	9.78±4.95	10.6±3.88	9.28±4.35	5.83±3.79	0.443	0.000**	0.079#
	Placebo group (n=15)	4.67±2.38	5.87±2.20	6.47±3.44	6.60±3.44	9.33±4.79	9.07±4.83	8.07±5.04	7.80±4.51			
Total Nasal Symptom Score (TNSS)	DGLA group (n=18)	5.22±4.08	5.00±3.65	4.94±3.44	5.17±3.88	6.06±3.42	6.61±2.87	6.06±3.51	3.61±2.77	0.588	0.000**	0.083#
	Placebo group (n=15)	3.33±2.02	4.27±1.75	4.53±2.26	4.53±2.26	6.00±3.68	5.80±2.96	5.27±2.99	5.07±2.84			
Total Ocular Symptom Score (TOSS)	DGLA group (n=18)	2.17±1.89	2.28±1.78	2.22±1.70	3.00±1.91	3.72±2.02	4.00±1.64	3.22±1.93	2.22±1.59	0.340	0.000**	0.309
	Placebo group (n=15)	1.33±1.23	1.60±1.06	1.93±1.33	2.07±1.58	3.33±1.88	3.27±2.19	2.80±2.40	2.73±1.91			

**$p < 0.01$,#$p < 0.1$.
